# Gaze Behavior of Referees in Sport—A Review

**DOI:** 10.3389/fspor.2020.572891

**Published:** 2020-11-10

**Authors:** Gal Ziv, Ronnie Lidor, Sima Zach, Stephanie Brams, Werner F. Helsen

**Affiliations:** ^1^The Academic College at Wingate, Wingate Institute, Netanya, Israel; ^2^Movement Control & Neuroplasticity Research Group, Department of Movement Sciences, KU Leuven, Leuven, Belgium

**Keywords:** gaze behavior, visual search, referees, umpires, perceptual-cognitive expertise

## Abstract

The purpose of this review was to examine the literature on gaze behavior in referees. A literature search found only 12 relevant studies. Five of those studies were conducted on referees in association football (soccer), three on judges in gymnastics, one on softball umpires, and one each on referees in team handball, rugby, and ice hockey. Seven studies reported differences in gaze behavior between referees of a higher skill level and those of a lower skill level, while four studies found no differences. In addition, five studies reported differences between referees of different skill levels in both gaze behavior and performance, while four studies found differences in performance only. A number of methodological concerns arise from the current review. Among them are the lack of studies conducted in ecologically valid conditions, the lack of studies on peripheral vision, and the lack of data on referees who are working together as teams. Based on this review, we conclude that additional research is needed to clarify the relationships between gaze behavior and performance in refereeing. Practitioners who work with referees should be cautious when adopting gaze training strategies to improve selective attention, since the data on their effectiveness are scarce and sometimes contradictory.

## Introduction

To increase the chances that the best athlete or team will win competitions or games, referees (or—as they are also called—judges, linesmen, officials, or umpires, depending upon the sport) have an official position in most of competitive individual and team sports, in order to ensure that the rules of the sporting event at hand are being applied (Bar-Eli et al., 2011). The perceptual–cognitive demands imposed on referees differ across sports, mainly due to the specific characteristics and nature of each sport. For example, the requirements of referees in association football (soccer) are different than the requirements of judges in gymnastics. However, regardless of the nature of the sport, referees are frequently required to make decisions under time constraints and emotional stress in challenging and dynamic settings.

To make accurate decisions, referees are usually required to search visually for relevant information in the sporting environment (Helsen and Bultynck, 2004). More specifically, they must attend to the most relevant environmental cues (e.g., the performer, an object related to the sport or game) while ignoring irrelevant information that may interfere with their decision making. Usually, the referee has just one chance to make the correct decision, as the observed act is performed only once. However in a number of sports referees are currently able to utilize advanced technology - such as the Video Assistant Referee (VAR) technology used in association football - to reexamine their decisions in certain game situations. However, in most sports these technological aids are not available to assist the referee. Therefore, the ability of the referee to effectively attend to the relevant information at each moment of the competition/game is crucial for achieving a high level of proficiency in his or her decision making (MacMahon et al., 2007).

Understanding how referees guide their visual search can increase our knowledge on how they perceive their sporting environment. For example, referees might fail to perceive cues in the environment that are salient to the spectators. If the visual search was guided to a certain environmental cue, it can be understood why another cue in the visual field was disregarded. Indeed, the relationship between gaze and performance is of major importance in sport (for a review, see Brams et al., 2019). In most circumstances, gaze behavior closely corresponds to the distribution of visual attention and visual processing across the visual field (eg., Henderson, 2003). Therefore, it can be argued that specific gaze patterns can lead to improved performance by enabling more appropriate information processing (see, Brams et al., 2019).

For the purpose of our review, we define gaze behavior as the eye movements referees perform in a sport context that are relevant to their officiating task. For example, assistant referees in association football (soccer) can direct their gaze toward the receiving player or toward the passing player when judging offside situations; gymnastics judges need to gaze at the gymnasts' body parts and at the scoreboard; and baseball and softball umpires standing behind the plate need to direct their gaze at an invisible area that represents the strike zone.

Researchers can use different variables to measure the four main aspects of eye movements: (a) the location of fixations—the relevant cues to which the referee directs his or her gaze, (b) the duration of fixations—how long the referee focuses on those cues, (c) the timing of fixations—the point in time at which the referee starts fixating on a certain cue, and (d) the order of the visual scan—whether the referee shifts his or her gaze from one cue to another in a structured or in a random manner. Each of these variables, as well as the relationships between them, is important for our understanding of the referee's process of decision making.

These variables usually measure central, or foveal, vision. However, extraction of visual information is not limited to foveal vision. While the fovea—which represents about 5° of the visual field and has the highest visual acuity (Millodot, 2017)—allows us to clearly see visual stimuli, we also rely on peripheral vision for visual processing (Rosenholtz, 2016). Peripheral vision allows, for example the tracking of multiple objects and the detection of changes in the environment that require a response (Vater et al., 2017). Research on gaze behavior should address both foveal and peripheral vision and take into consideration that referees in different sports need to attend to different visual cues in the visual field in order to perform adequately (see Table 1).

**Table 1:** A description of environmental constraints, referees' tasks, and location of relevant cues in the visual field across the sports reviewed.

**Sport**	**Environmental constraints**	**Primary tasks**	**Location of relevant cues in the visual field**
Association Football (assistant referees)	Dynamic environment. The assistant referee is required to constantly adjust his/her position in order to observe relevant environmental cues.	Identify off-side situations. Identify foul plays.	Calling an offside correctly requires both foveal and peripheral vision.
Softball	Umpires mostly stationary. Some dynamic and some relatively static plays.	Call ball/strike behind the plate. Call safe/out around the bases.	Mostly foveal vision.
Gymnastics	Judges are stationary. Usually, a number of gymnasts perform at the same time.	Follow specific body movements. Score accuracy of performance.	
Association Football (referees)	Highly dynamic and fast sport. Referees must anticipate the action. Referees must avoid being “ball watchers.”	Identify foul plays and other game-play violations.	Calling a foul requires anticipation, advance cue usage, and pattern recognition. Peripheral vision to direct attention to possibly relevant action away from the puck or ball. Foveal vision to identify foul plays and other violations.
Ice Hockey	Highly dynamic and fast sport. Referees must anticipate the action.		
Rugby	Dynamic and stationary acts. The referee must move with the action, but there are times when the players are mostly in one location.		
Team Handball	Moderately dynamic—mostly played with a set offense and a set defense, but with fast transitions in-between.		

As far as we know, there are currently no review articles on the gaze behavior of referees in sport. This is despite the influx of research on sport officiating in the past two decades (for a review, see Hancock et al., 2020). Therefore, in the current article we review a series of studies (*N* = 12) focusing on gaze behavior in this population. In this review, we (a) discuss and compare evidence-based knowledge about gaze behavior in referees of different skill levels (e.g., national and international referees, experts, and non-experts) and in different sports (team and individual); (b) discuss a number of methodological concerns and research limitations; and (c) propose several ideas for additional studies on gaze behavior in referees. In addition, we argue that due to the small number of studies and their limited findings, we should adopt a cautious approach when implementing these findings in training programs aimed at improving gaze behavior in referees.

## Literature Search

We conducted an electronic search of the literature in two databases—EBSCO Discovery Service and Google Scholar. For the search in EBSCO Discovery Service, we used the following search terms: (referee^*^ OR judge^*^ OR umpire^*^ OR official^*^) AND (gaze OR visual search OR visual attention OR perceptual–cognitive OR eye movements) AND (sport^*^ OR basketball OR baseball OR handball OR volleyball OR gymnastics OR soccer OR football OR hockey OR tennis OR diving). We limited the search to peer-reviewed articles published in the English language. Only studies that measured gaze variables were included. We excluded studies that reported gaze instructions but did not record gaze. For the search in Google Scholar, we searched for a combination of the following terms in the title only: *referee, official, umpire, gaze behavior*, and *visual search*. In addition, the lists of references from relevant studies were scanned manually for further sources. The search was performed in March, 2020.

**Figure 1:**
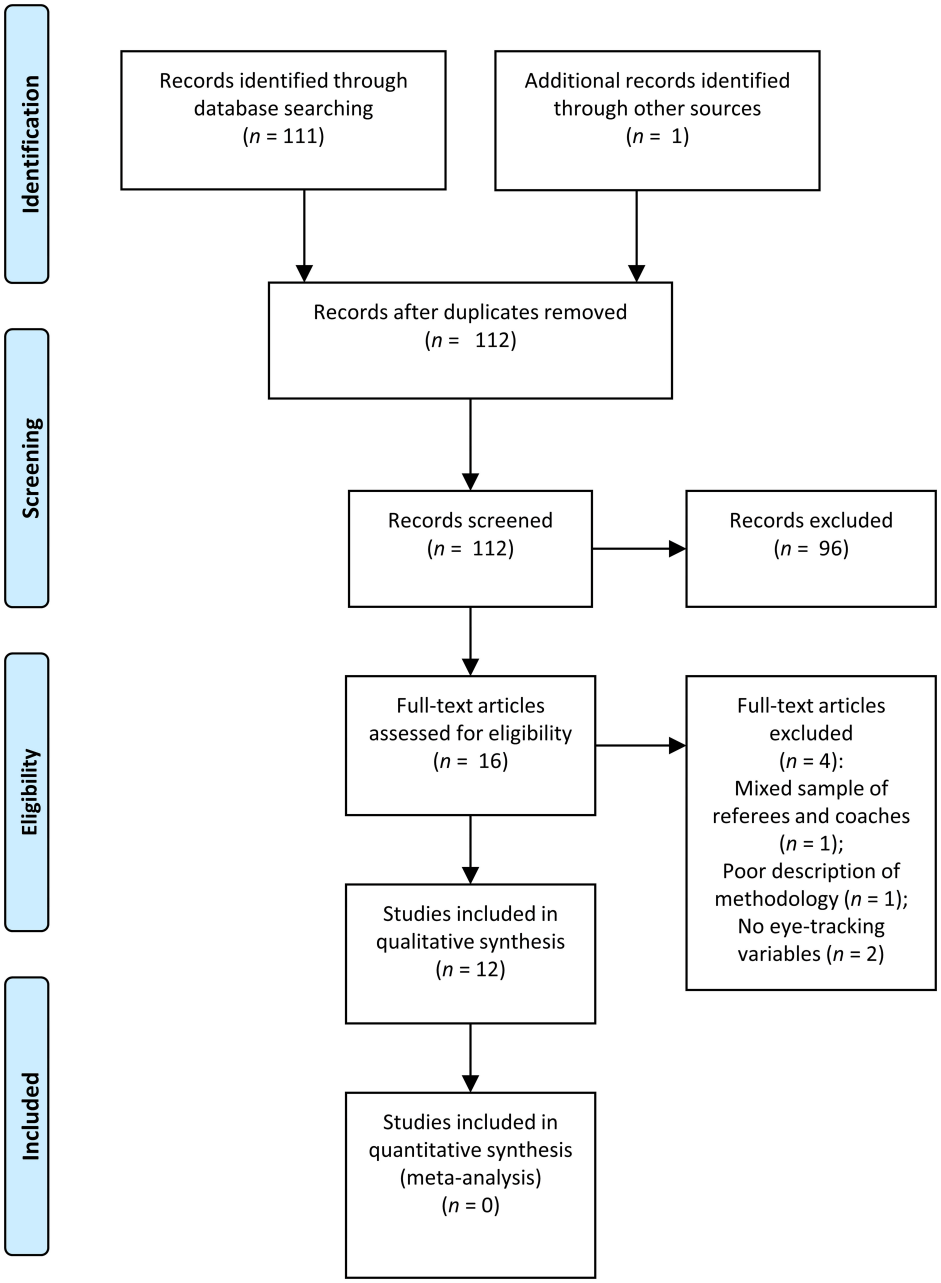
PRISMA flow diagram of the article selection process.

A flow diagram of the study selection process based on the PRISMA Statement (Liberati et al., 2009) is presented in Figure 1. The electronic search yielded 11 relevant studies and the manual search yielded one more study, for a total of 12 studies (Bard et al., 1980; Catteeuw et al., 2009, 2010b; Hancock and Ste-Marie, 2013; Millslagle et al., 2013; Flessas et al., 2015; Luis et al., 2015; Spitz et al., 2016; Schnyder et al., 2017; Fasold et al., 2018; Pizzera et al., 2018; Moore et al., 2019).

## Gaze Behavior of Referees in Various Sports

The findings of the reviewed studies are summarized in Table 2. Out of the 12 reviewed studies, five were conducted on referees in association football (soccer), three on judges in gymnastics, one on softball umpires, and one each on referees in team handball, in rugby, and in ice hockey. Seven studies reported differences between referees of a higher skill level and those of a lower skill level in gaze behavior, while four studies found no differences. Table 3 lists the studies that found differences between different levels of referees in gaze behavior only, in performance only, or in both gaze behavior and performance. Indeed, the reviewed studies examined gaze behavior in referees from different sports, and presumably the referees acted under different environmental constraints. However, because only a small number of studies were found (12), we preferred to synthesize the research findings that emerged from all the reviewed studies in spite of the differences in the officiating environments. Therefore, we also took into account these differences while discussing the findings.

**Table 2:** A summary of studies (*N* = 12) examining gaze behavior of referees in sport (ordered by year of publication).

**References**	**Sport**	**Participants**	**Procedure**	**Gaze behavior**	**Performance**
Bard et al., 1980	Gymnastics	Canadian nationally certified judges (*n* = 4, 4–8 years of experience), local judges (*n* = 3, 1 year of experience)	Evaluate four video-recorded routines on a balance beam Performance compared to an expert judge who watched the routines in slow motion	# of fixations: National < local (by 27%, not statistically significant—small sample size) Fixations on upper body: National > local Fixations on legs: National < local	# of judgment mistakes: National < local (by 50%, not statistically significant—small sample size, large variability in the local judges)
Catteeuw et al., 2009	Association football	International (*n* = 5) and national (*n* = 5) ARs	Judging 40 videotaped simulated offside situations recorded from the perspective of an assistant referee Four categories of movies: more >1 m behind the offside line, <1 m behind the offside line, same line as offside line, <1 m ahead of offside line	# of fixation and mean fixation duration per video: International = national Greatest amount of time spent fixation the offside line After the pass: International referees spent more time fixation the receiving attacker	Offside decision accuracy: International > national Bias toward flag errors in national referees only Highest error % when attacker on offside line (42% international, 72% national)
Catteeuw et al., 2010b	Association football	Exp. 1: Elite ARs (*n* = 17) divided by median split to successful and less-successful based on offside decision accuracy	Exp. 1: Similar to Catteeuw et al., 2009 with the addition of the selection of the exact location of the attacking receiver from five possible images	Exp. 1: # of fixations, fixation duration, percentage viewing time on areas of interest: Offside line > passer No differences between groups	Exp. 1: Total accuracy: 75% Successful−82.2% Less-successful−64.4% Bias toward flag errors in less-successful only Accuracy in memory of frame recognition: Successful more consistent, less successful—more bias forward
		Exp. 2: Training group: elite FIFA ARs (*n* = 10) Control group: National referees (*n* = 14)	Exp. 2: Pre-test and post-test like Exp. 1 Training group received four training interventions for a total of 80 video simulations and 80 computer animations of offside situations	Exp. 2: For training group only: no differences between pre- and post-test	Exp. 2: Training grouped improved from pre-test to post-test (from 71.3 to 78% correct decisions) Control group did not improve (~63% correct) No bias toward flag errors in both groups Training group improved consistency in memory of exact location of attacker
Hancock and Ste-Marie, 2013	Ice hockey	Higher-level (*n* = 15) and lower-level (*n* = 15) referees	Decide on infractions (e.g., hooking, tripping, roughing, elbowing) when watching 26 video clips of 3–6 s on a computer	No differences between higher-level and lower-level referees	Decision accuracy and sensitivity: Higher-level > lower-level
Millslagle et al., 2013	Fast-pitch softball	Experienced (*n* = 4, >10 years behind the plate), and inexperienced (*n* = 4, <1 year behind the plate) varsity, college, and university umpires	Calling ball or strike for 10 fastballs and 10 change ups when standing behind the plate The hitter either swung or did not swing to create the experience of a real game situation	Total fixations/saccades/blinks: Experienced < inexperienced Ball-tracking duration: Experienced > inexperienced Fixation duration on ball release area: Experienced > inexperienced Onset of fixation on ball release area: Experienced earlier than inexperienced	N/A
Flessas et al., 2015	Rhythmic gymnastics	International (*n* = 10), national (*n* = 10), and novice (*n* = 10) level judges	Judge two videos of a five-gymnast ensemble: with a hoop and with ribbons and ropes	Time looking away from the video: Novice < international, national Mean time eyes overlaid the reported vs. unreported errors: Novices:	Judging performance: International > national, novice All judges reported <40% of the errors False alarm rate: National > international, novice
				Reported > not reported International and national: Reported = not reported Eye position affected performance only in national judges	
Luis et al., 2015	Association football	Referees (*n* = 8) with 6 years of experience refereeing regional leagues and lower national leagues	Judging 24 videotaped offside situations from three distances and from different viewing angles between passing and receiving attacking players (under and over 35°)	Gaze moved from player with ball to receiving player and last defender as time progressed toward the pass # and duration of fixations on player with ball just before pass: Large view angle > small view angle At larger distances: increased # and duration of fixations on receiving player At a shorter distance: increased # and duration of fixation on middle area (between passer and receiver)	Accuracy: Small view angle > large view angle At smaller view angles—more errors from far distances At larger view angle—more errors from near distances
Spitz et al., 2016	Association football	Elite (*n* = 20, 16 years of experience, 9 years at professional level), and sub-elite (*n* = 19, 12 years of experience—not in professional level)	Judging 20 foul play in open play (1 vs. 2 or 2 vs. 2) and in corner kicks (6–7 vs. 6–7, 1 goalkeeper) on video filmed from 1st person perspective of an assistant referee Making technical decisions (e.g., free kick, penalty kick) and disciplinary decisions (e.g., yellow, red card)	# and duration of fixations: Open play and corner kicks: Elite = sub-elite Fixation location: Open play: Time fixating contact zone: elite > sub-elite Corner kicks: Time fixating contact zone of attacker: elite > sub-elite (*p* = 0.07)	General accuracy: Open play < corner kicks Open play: Technical decision accuracy: Elite = sub-elite Disciplinary decision accuracy: Elite > sub-elite Corner kicks: Technical decision accuracy: Elite > sub-elite Disciplinary decision accuracy: Elite = sub-elite
Schnyder et al., 2017	Association football	Expert (*n* = 3, FIFA license, international experience) and near-expert (*n* = 3, national experience) ARs	Judging 36 offside scenes performed by an under-21 team in a real field with three attackers, three defenders, and a goalkeeper	Fixation locations: Experts = near-experts	Correct decisions: Expert > near-expert In doubtful situations—tendency of both groups to keep the flag down More correct decisions when offside line was fixated at the time of the pass and when this fixation was longer
Fasold et al., 2018	Team handball	A case study of a team of two referees with 6 years of experience at the regional level	Refereeing a real training match of two semi-professional teams from the two required positions: field referee and goal referee Analysis of set offense and defense Analysis of 44 attacking scenes (mean 6.7 s each) in total	Fixating action with ball: In 75% of cases, both referees fixated the same area and mostly the area of action with the ball. But: Field referee > goal referee When referees fixated on different aspects of the game (only 15% of time): When the goal referee fixated on action with ball: Field referee fixated on action without ball ~75% of time, the other referee ~15% of time When field referee fixated on action with ball: Goal referee fixated on action without ball ~81% of time, the other referee ~15% of time	N/A
Pizzera et al., 2018	Gymnastics	High-level (*n* = 15) and low-level (*n* = 20) gymnastics judges with or without specific motor experience as an athlete in gymnastics	Judge videos of handsprings forward with a half turn on/half turn off on a vault Each video lasted ~4.27 s	# of fixations: High level > low level for whole skill and for landing phase With motor experience > without, for whole skill # of Fixations on head and arms: High level > low level # of Fixations on legs: With motor experience > without Fixation duration: With motor experience > without	Judgement quality: High level > low level With motor experience = without
Moore et al., 2019	Rugby	Elite referees from highest division of professional rugby (*n* = 9), trainee referees from a university-based academy, refereeing lower competitive levels (*n* = 9), experienced rugby players who never refereed (*n* = 9)	Decide on possible infractions while watching 10 videos of scrum situations (5–25 s in duration) on an 83-inch screen Four possible decisions: Play on, reset, penalty attacking team, penalty defending team	Search rate (fixations·s^−1^) Elite < trainee < player Entropy: Elite, trainees < players Percentage viewing time: Central-pack: Elite, trainee > players outer-pack, and non-pack: Elite, trainee < players	Decision-making accuracy: Elite, trainees > players Percentage viewing time and accuracy contributed to a significant proportion of the variance in decision-making accuracy

**Table 3:** Studies that reported differences between referees with different skill levels in performance, gaze behavior, or both (*n* = 11)^*^.

**Differences in performance only**	**Differences in gaze behavior only**	**Differences in both performance and gaze behavior**
Catteeuw et al., 2009^#^–Association football	Millslagle et al., 2013^**^–Softball	Spitz et al., 2016—Association football
Catteeuw et al., 2010b—Association football	Fasold et al., 2018^**^–Team Handball	Bard et al., 1980—Gymnastics
Schnyder et al., 2017—Association football		Pizzera et al., 2018—Gymnastics
Hancock and Ste-Marie, 2013—Ice Hockey		Flessas et al., 2015—Rhythmic Gymnastics
		Moore et al., 2019—Rugby

* *The referees in the study by Luis et al. (2015) were of the same level, and therefore 11 of the 12 reviewed studies are included here*.

** *Did not report performance variables*.

# *This study was placed here despite the reported difference in fixation location after the pass occurred in an offside decision-making task in association football. We consider that this difference may not have affected performance, as all other gaze variables were similar between the international and national referees*.

### Association Football

We found four studies that examined the ability of assistant referees (ARs) to judge whether a player was on- or off-side (Catteeuw et al., 2009, 2010b; Luis et al., 2015; Schnyder et al., 2017), and one study that examined the ability to identify foul plays (Spitz et al., 2016). In one of these studies (Catteeuw et al., 2009), five international and five national ARs were requested to judge 40 recorded videos of simulated possible offside situations. Out of the 40 videos, 10 had the attacker at least one meter behind the offside line, 10 had the attacker <1 m behind the offside line, 10 had the attacker on the offside line, and 10 had the attacker <1 m ahead of the offside line. The international ARs arrived at more correct decisions compared to the national referees (83.5 vs. 74.5%, respectively), despite showing a similar gaze behavior. In fact, both groups of referees mostly fixated on the offside line. The only difference in gaze behavior between the groups of ARs was that after the pass, the international ARs tended to shift their gaze to the receiving attacker whereas the national ARs tended to maintain their gaze on the offside line. It is possible that the international referees were able to assess the position of the receiving attacker at the time of the pass from gazing at him or her immediately after the pass.

Findings also showed that national ARs demonstrated a bias toward calling an offside erroneously, while the international ARs did not show this tendency. This bias was also reported in previous studies (e.g., Gilis et al., 2009). One possible explanation for such a bias is a perceptual illusion called the *flash-lag effect* (Nijhawan, 1994), in which a moving object can be perceived ahead of its actual position at a certain instant that is usually expressed by a brief marker. In offside situations, the receiving attacker may be perceived ahead of his or her actual position at the instant of the pass. Because gaze behavior did not differ between the referees, it is possible that international ARs—perhaps based on their greater experience—possess more optimal cognitive strategies to correct for this bias.

Similarly to the earlier Catteeuw et al. (2009) study, Catteeuw et al. (2010b, Exp. 1) showed no differences in gaze behavior but did reveal a bias toward raising the offside flag by less-successful referees but not successful referees (divided by a median split of decision accuracy). In Exp. 2 of their study (Catteeuw et al., 2010b), a group of referees was trained to make offside decisions by watching 80 video simulations and 80 computer animations of offside situations. Compared to the referees in a control group who did not improve their accuracy from a pre-test to a post-test, the referees who were trained did improve their decision accuracy. However, there were no differences in gaze behavior from pre- to post-testing.

Differences in offside decision accuracy and no differences in gaze behavior between expert and near-expert ARs were also reported by Schnyder et al. (2017). In this study, three expert ARs made more correct decisions (91.4%) than three near-expert ARs (79.8%) when judging 36 offside scenes on a real field of play. There were no differences in gaze behavior between the two groups of referees. However, in both experimental groups, despite the small sample size, it appeared that a fixation on the offside line at the time of the pass (and preferably a longer fixation) resulted in a higher number of correct responses.

The distance of the AR and the size of the visual field between the passing and the receiving attacker may affect performance and gaze behavior as well. Indeed, in one study of eight intermediate-level ARs (Luis et al., 2015), offside decision accuracy was higher when the viewing angle was under 35° compared to over 35°. In addition, at a higher angle more errors were made at a short distance, whereas at a smaller angle more errors were made at a long distance. It is possible that when the distance is short and the viewing angle is large, more saccades of larger amplitudes occur when attempting to see both the passing and the receiving players, and it is easier to fixate on the wrong location. At larger viewing angles, more fixations of longer durations were made to the passing player immediately before the pass. In addition, more fixations of longer durations were directed to the middle area between the passing and the receiving attacking players. This may represent an attempt to use peripheral visual information from both the passing player and the offside line.

Finally, one study (Spitz et al., 2016) examined gaze behavior and decision accuracy of elite and sub-elite referees when judging videos of foul-play situations during open play and during corner kicks. In each situation, the referees were asked to make a technical decision (e.g., penalty kick, free kick) as well as a disciplinary decision (e.g., yellow, red, or no card). During open play, elite referees were much more accurate than sub-elite referees in making disciplinary calls (61 vs. 45.3%, respectively) but not technical calls (54.5 vs. 49.5%, respectively). In contrast, during corner kicks, elite referees were more accurate than sub-elite referees in making technical calls (69.5 vs. 56.8%, respectively) but not disciplinary calls (82.5 vs. 82.6%, respectively). There were no differences between the two groups in the number and duration of fixations. However, compared to sub-elite referees, elite referees spent more time fixating on the contact zone of the attacker in open play situations and also spent more time (although only nearing significance) fixating on the contact zone in a corner kick situation. The results of this study suggest that gaze behavior of referees is related to their decision accuracy in assessing foul play.

In summary, it appears that in offside situations the difference in decision-making accuracy between groups of referees is not related to the foveal visual information (constituting the area of maximum visual acuity and color discrimination) they attend to and that ARs should fixate on the offside line at the time of the pass. The lack of difference in gaze behavior between different levels of referees, and the higher decision accuracy in expert referees, suggest that the expert referees use other perceptual–cognitive strategies for their decisions. For example, Larkin et al. (2018) used qualitative methodology to evaluate three Australian Football umpires and observed three themes related to cognitive processes and decision making: knowledge of game-play, player intention during game-play, and decision evaluation—the process that leads to the actual decision. In addition, Spitz et al. (2018) reported that, compared with sub-elite referees, elite referees were better at anticipating (by using advance cues) and recalling foul plays. It is also possible that expert referees are better at extracting relevant information from their peripheral vision; this should be examined in future research. The use of peripheral vision may be of greater importance for ARs' decisions as the viewing angle between the passing and the receiving player increases. However, these suggestions may not be accurate when foul-play decisions are being made, since the one study on this topic found differences in gaze behavior between referees of different skill levels.

### Softball

One study examined the gaze behavior of behind-the-plate umpires in fast-pitch softball (Millslagle et al., 2013). Compared to the four inexperienced umpires who participated in this study (<1 year of experience calling pitches behind the plate), the four experienced umpires (>10 years of experience) showed a more economical gaze behavior (i.e., lower number of fixations, saccades, and blinks), earlier onset and longer duration of fixations on the ball release area, and longer tracking of the ball in flight. Unfortunately, accuracy in calling strikes or balls was not reported in this study, and therefore it is not known whether the differences in gaze behavior were related to the umpires' performance.

Calling a strike or a ball accurately and consistently is a challenging perceptual judgment (DeLong, 2017) and is crucial in the game of softball or baseball. Umpires in Major League Baseball are mostly accurate and, at least according to one source (Moskowitz and Wertheim, 2011), make erroneous strike or ball calls only 14.4% of the time. This level of accuracy is impressive, given the ball's velocity and spin and the very short time it takes to reach home plate. As the decision making behind the plate is a classic perceptual–cognitive task based on visual information, we propose that it should be investigated further. Understanding the gaze behavior of expert baseball umpires may allow this knowledge to be incorporated into gaze training for umpires.

### Gymnastics and Rhythmic Gymnastics

We found three studies examining gaze behavior of judges in gymnastics and rhythmic gymnastics (Bard et al., 1980; Flessas et al., 2015; Pizzera et al., 2018). Bard and colleagues found no statistically significant differences between four experienced and three less-experienced judges. However, due to the large effect sizes, these differences may be meaningful. More specifically, the experienced judges made fewer total fixations (82) compared to the less-experienced judges (112). In addition, the experienced judges directed their gaze toward the head and arms of the gymnast more often than the less-experienced judges (31.5 vs. 14% of all fixations, respectively). This trend was reversed for gazing at the gymnast's legs (17.5 vs. 27.5%, respectively). In addition, the experienced judges made fewer errors (~12) compared to the less-experienced judges (~21).

In another study (Pizzera et al., 2018), 35 gymnastics judges were asked to judge video clips of vault exercises. The judges were divided into high- or low-level, based on their official judging license as well as on whether they had motor experience in performing vault exercises. Similar to Bard et al. (1980), high-level judges made more fixations on the head and arms but also made more total fixations. This was accompanied by improved judging performance. However, judges with specific motor experience made more fixations on the legs compared to those who did not have any motor experience. Those with motor experience also presented longer fixation durations compared to those without motor experience. These differences did not materialize into differences in judging performance.

A third study (Flessas et al., 2015) examined the performance and gaze behavior of international, national, and novice rhythmic gymnastics judges who judged videos of a five-gymnast ensemble performing two exercises (hoops, ribbons and ropes). International judges performed better than national and novice judges. However, the judges' performance was relatively poor, as international judges reported ~40% of the performance errors and national and novice judges reported ~20% of the errors. As the authors suggested, these results are not surprising as the task of keeping track of five gymnasts at one time is a difficult one. As for the judges' gaze behavior, novices spent more time gazing at the errors that they eventually reported compared to the errors that they missed. In contrast, there was no difference between the time international judges and national judges gazed at reported and missed errors. This finding suggests that due to their inexperience, novice judges may need more time to process an error, and therefore they have a higher chance of missing errors that occur simultaneously at different locations. Unlike novice judges, who were not efficient at detecting errors (i.e., longer fixations on errors yet a lower probability of reporting errors), national judges showed a much higher efficiency (i.e., a higher proportion of fixated errors were reported). However, the international-level judges did not show such efficiency. As the authors suggested, this finding may suggest that international judges rely on other cognitive processes to detect errors. It is possible, for example that (a) their experience allows them to use much shorter fixation durations to assess errors, (b) they may rely more on peripheral vision, and (c) they may be better at anticipating and detecting errors.

In summary, the findings that emerged from the three abovementioned studies suggest that gaze behavior is related to judging performance in gymnastics but that this relationship is less apparent in rhythmic gymnastics. Specifically, it appears that more experienced judges make more fixations in general, and specifically they fixate their gaze to the head and the arms. However, it remains to be seen whether this gaze behavior can be taught and therefore lead to improved judging performance. For acquiring this instructional knowledge, gaze-training studies are required.

### Ice Hockey

We found one study that examined gaze behavior and decision making in ice hockey referees (Hancock and Ste-Marie, 2013). High-level and low-level referees (the higher-level referees were recruited from Junior and Under-18 AAA leagues, while the lower-level referees were recruited from youth ice hockey) were asked to make decisions about infractions while watching 3- to 6-s video clips. While the higher-level referees showed greater accuracy and more sensitivity compared to the lower-level referees, gaze behavior did not differ between the two. These results suggest that, compared to lower-level referees, higher-level referees use cognitive processes that allow them to extract and process relevant visual information. It is possible, for example, that higher-level referees can more effectively compare what they see to what is stored from experience in their long-term memory (Ericsson and Kintsch, 1995).

However, other explanations for these findings are noteworthy. First, as the authors of this study (Hancock and Ste-Marie, 2013) argued, gaze behavior when looking at a 30-inch screen differs from gaze behavior in real situations. Indeed, it is likely that the larger visual field in a real game requires more dynamic gaze and eye movements (see, for example, Gegenfurtner et al., 2011). Second, the participants in this study were not expert referees. It is possible that differences in gaze behavior would have been noticed if expert referees from the National Hockey League had been assessed in this study. Finally, the gaze variables in this study were rather general (i.e., number of fixations, average fixation duration). It is possible that differences between the referees would have been found if measures of visual search relating to specific areas of interest had been analyzed. For example, it would be interesting to know if the two groups of referees spent their time gazing at specific bodily areas differently (e.g., fixating more or less on the torso, hands, or head), or at the hockey stick. Finally, it is possible that the structure of the visual search (i.e., a more structured vs. a more random visual search) differed between the referees. Additional studies should use gaze variables, such as these, because they may shed more light on the underlying processes of expert judgment in ice hockey.

### Rugby

One study examined gaze behavior and performance of elite referees, trainee referees, and players in rugby (Moore et al., 2019). When watching videos of scrum scenarios, elite and trainee referees made more accurate judgments (53–58% accuracy) compared to the rugby players (39%). The similar judgment accuracy between elite and trainee referees can be explained by the relatively substantial experience (~4 years) acquired by the trainee referees.

Compared to the rugby players, elite and trainee referees had lower search entropy values (indicating a more structured search) and had a higher percentage of viewing time toward the central-pack and a lower percentage of viewing time toward the outer-pack and to non-pack areas. The one gaze variable that differed between elite and trainee referees was the search rate. Elite referees made fewer fixations per second compared to trainee referees, while both groups made fewer fixations per second compared to the players.

This finding is in contrast to the findings of Hancock and Ste-Marie (2013), who found no differences in gaze behavior between higher-level and lower-level referees in ice hockey. One possible explanation for this difference is the variations in the size of the monitor on which the videos were played (30 vs. 83-inch for Hancock and Ste-Marie, 2013; Moore et al., 2019, respectively). Another possible explanation is that search rate is a more sensitive measure for differences between levels of expertise, and this measure was not used by Hancock and Ste-Marie (2013). As Moore et al. (2019) suggested, it is also possible that the differences in gaze behavior are due to differences in task requirements. Indeed, scrum scenarios in rugby are rather static, while ice hockey plays are more dynamic. Lastly, it is worth noting that the 83-inch screen in Moore et al. (2019) study allows for a better representation of the actual refereeing task because it allows for more realistic viewing angles and, as such, is better for assessing the relationship between perception and action in sport (Dicks et al., 2009).

### Team Handball

In our search, we found one case study that examined gaze behavior of two team-handball referees during a training game (Fasold et al., 2018). Among the 12 studies discussed in our review, this study is the only one that attempted to examine shared gaze behavior of the referees. The results of the study showed that the referees mostly fixated on the same area in the field of play, and this was usually where the action with the ball took place. In only about 15% of the cases did one referee fixate on the area of action with the ball, and the other referee fixated on the area of action without the ball. Unfortunately, the referees' decision-making accuracy was not evaluated, and therefore it is not known whether the team gaze behavior led to erroneous decisions. On the one hand, when both referees gaze at the same location, they may miss possible infractions that are taking place away from the ball. On the other hand, gazing at the same location can improve decision making when the situation is dynamic and rapidly changing.

Regardless, this case study shows that evaluating team gaze behavior is possible. This is important, since improved or optimal shared gaze behavior can lead to better decision-making processes in the dynamic situations that are often encountered by referees in various team sports. In such situations, it is usually not the individual referee that makes the correct decision. Rather, it is the team of referees that makes the correct decision. Therefore, it is the team's shared gaze, shared cognition, and shared decision making that should be studied.

## Methodological Concerns, Research Limitations and Future Research

Based on the reviewed studies, seven methodological concerns are discussed—six specific and one general. Each concern leads to ideas or principles that are relevant for carrying out additional studies on gaze behavior in referees in sport.

### Missing Theoretical Background

Both the “expert performance approach” (Ericsson and Smith, 1991) and the theories of visual search and visual attention (for reviews, see Wolfe and Horowitz, 2017; Wolfe, 2018; see also Gegenfurtner et al., 2011 for a somewhat different approach) are available in the literature for understanding the underlying processes related to visual search behavior and expertise. Explaining these theories is beyond the scope of this review. However, most of the reviewed studies fail to address these theories (Moore et al., 2019, is an exception). To further evaluate the underlying mechanisms related to visual search and expertise in referees, we suggest that future work should consider these theories when designing research and selecting gaze variables.

An example from the current review is noteworthy. In one of the reviewed studies (Moore et al., 2019), the authors addressed the possibility that their findings are in line with the information reduction hypothesis (Haider and Frensch, 1999). In general, this hypothesis suggests that experts make more fixations of longer durations to relevant areas in the visual field, and fewer fixations of shorter durations to areas that are irrelevant to the task at hand. Indeed, Moore et al. (2019) measured gaze to specific areas of interest and found differences between experts and novices. In contrast, another study (Hancock and Ste-Marie, 2013) did not report gaze differences between higher- and lower-level referees. However, the gaze variables used in this study did not allow for analyzing areas of interest in a visual field. Recording and analyzing appropriate gaze variables will allow researchers to assess whether their results are in line with a certain visual search theory. Specifically, the number and duration of fixations on relevant areas of interest, the scan pattern systematicity, and the measures of one's visual span (i.e., latency to the first fixation on a relevant area of interest and the length of saccade amplitudes) can provide meaningful information related to search theories (Gegenfurtner et al., 2011).

### Lack of Studies on Gaze Behavior of a Team of Referees

In most team sports (e.g., association football, basketball, ice hockey), it is a team of referees—rather than one referee—that is responsible for making accurate decisions. Consequently, it is primarily the team's coordinated gaze behavior that will lead to appropriate refereeing performances. We found only one study that examined gaze behavior of a team of two referees (Fasold et al., 2018). This study showed that the referees' joint gaze behavior mostly fixated on the same areas; however, we cannot determine whether this improves or obstructs decision making because the authors did not report decision accuracy. With the availability of relatively inexpensive and accurate wearable eye trackers, we suggest that conducting such research is timely and will greatly advance our knowledge on refereeing and decision making in team sports. These studies will also complement (and gain insights from) the available literature on team cognition or team coordination in sport (e.g., Eccles and Tenenbaum, 2004; Fiore and Salas, 2006; see also Silva et al., 2013, for an ecological dynamics approach). Finally, these studies also discuss the importance of shared cognition for the performance of a team of players, and insights from this literature can be extended to teams of referees.

### Lack of Studies on Peripheral Vision

The role of peripheral vision in expert performance has been examined in athletes in various sports (for a review of results and research methodologies on this topic, see Vater et al., 2019). Indeed, peripheral vision provides us with relevant visual information, even though visual acuity and color perception decline in the periphery (Rosenholtz, 2016). However, none of the studies in the current review examined peripheral vision. This is unfortunate, as much of the information that can be obtained from peripheral vision could be useful for referees. For example, it is probable that ARs who judge offside situations in association football use information from their peripheral vision. That is, these referees fixate on the offside line, and if the visual field is not large they may also perceive the passing player in their peripheral vision. We suggest that it is vision as a whole that leads to optimal perception, and not solely foveal vision. Indeed, a relationship between peripheral information processing and superior offside decision making was already reported by Hüttermann et al. (2018). Therefore, future studies should assess both foveal and peripheral vision in order to obtain a more complete understanding of the impact of gaze behavior on decision making in referees.

For example, researchers can study peripheral vision by blurring the video and allowing participants to see clearly only in their foveal visual field. Ryu et al. (2015) implemented this method to examine the contribution of peripheral vision and central vision in basketball. In their study, participants saw a clear image at a visual field of 2.5° around their fixation point, and the rest of the image was blurred. This viewing condition can then be compared to regular viewing conditions (no blur), and even to conditions that show peripheral areas clearly and blur central vision.

### Lack of Studies in Ecologically Valid Conditions

Out of the 12 reviewed studies, only three measured gaze behavior in real playing conditions: one in association football (Schnyder et al., 2017), one in fast-pitch softball (Millslagle et al., 2013), and one in team handball (Fasold et al., 2018). In the remaining nine studies, referees viewed videos on screens of different sizes. Viewing videos on TV or computer screens does not represent the actual task on the field. More specifically, under such task conditions the referee is usually seated—rather than standing, walking, or running as in actual competition/game conditions—and the visual field is much smaller, especially when the monitors are rather small. It has been argued that to study perception and action in sport, task design should be as representative as possible of the real conditions (Dicks et al., 2009). It is likely that more realistic scenes contain more details, which often generate background interferences as well. Under such conditions, the differences between expert and less-expert referees may be more pronounced. In this respect, Kredel et al. (2017) suggested that eye-tracking studies strive, among other things, to include realistic viewing conditions and naturalistic responses on the one hand and robust measurements on the other hand. However, achieving accurate and robust eye-tracking measurements in ecologically valid conditions can be difficult. Here, like in other research domains, researchers are faced with a choice between external and internal validity (Kredel et al., 2017).

We suggest that researchers should try to mimic actual conditions as much as possible when studying the performance of referees. This can be done on a real field or by using virtual reality (VR) with high functional fidelity. While data on the transferability of VR to real-life situations are limited, recent studies have shown that training athletes for decision making in a VR environment can be generalized to the real game (Pagé et al., 2019). Therefore, VR is worth further examination. We also suggest that researchers should project videos on large screens that represent, as closely as possible, the actual field of view of the referee in a real match. Researchers should also film such videos from the correct first-person position of the referee in a match. Paying attention to such details can improve the ecological validity of video-based studies.

In this respect, two video-based studies are noteworthy. Catteeuw et al. (2010a) showed that off-field offside decision-making training using video clips and computer animations should be considered as part of training because they help the referees to gain more experience and to improve overall decision-making performance on the field. Another study (Put et al., 2013) showed that perceptual–cognitive skill training using a web-based application results in a positive and direct transfer to on-field offside decisions. It was argued that the structure and content of the web-based training intervention mimicked the perceptual difficulties of real-match situations and therefore helped the assistant referees to mediate and enhance their offside decision-making skills, both on- and off-field. While eye movements were not recorded in either of the studies, their results showed that video-based and online training may be beneficial for performance in ecologically valid conditions.

### Lack of Qualitative Studies

A recent review of sport officiating by Hancock et al. (2020) showed that most studies used quantitative methodologies (e.g., 78.5% of studies from 2010 to 2018). Similarly, all studies included in the current review used quantitative methodologies. Quantitative analyses are appropriate for measures of gaze behavior, but incorporating qualitative methodologies can improve our understanding of the complex decision-making tasks that referees face (see, e.g., Larkin et al., 2018). We can ask referees what they usually attend to in certain situations, whether they have specific visual strategies, how they work together to attend to most relevant occurrences in the field of play, etc. In addition, qualitative analyses may allow researchers to assess how referees process and interpret visual information.

### Gaze Behavior, Cognitive Strategies and Decision Making

While gaze behavior allows referees to attend to relevant stimuli, many other factors interact and lead to better or worse decisions. Fatigue, stress, physical fitness, environmental conditions, experience, anticipation, knowledge of game-play, and the player's intentions (Larkin et al., 2018) are some of the factors that, in addition to the referees' gaze behavior, will lead to a specific decision. The fact that several cognitive processes, physical fitness, and environmental conditions interact with gaze behavior and lead to good (or bad) decision making may explain the contradictory results in the reviewed studies. It is possible that with similar gaze behavior and attention to visual stimuli, expert referees use certain cognitive processes to better advantage than less-expert referees. Hence, future studies should use a more holistic approach and study gaze behavior alongside cognitive process, physical constraints, and environmental constraints.

### Other General Methodological Concerns

Several of the reviewed studies used relatively small sample sizes and did not report either the statistical power or corrections for multiple comparisons. While it can be difficult to recruit enough referees to meet the required statistical power, researchers should always correct for multiple comparisons. In addition, the variety of aims and dependent variables restricts our ability to generalize referees' gaze behavior. Researchers who wish to examine the relationship between gaze behavior and decision making in referees should first decide on their objective (e.g., training study, observational study); clearly choose the methodology that will allow proper statistical power (e.g., a repeated-measure design may improve statistical power when the researcher knows that the sample size will be small); and choose the relevant independent and dependent variables [e.g., what are the important variables—fixations? saccades? dwell times on a specific area of interest (AOI)?]. Then, researchers should assess whether they have access to the required eye tracking hardware and software, e.g., if saccades and micro-saccades are important, a 30- or 60-Hz eye tracker may not be sufficient, but for saccades larger than 5°, 200 Hz is sufficient (Andersson et al., 2010). Finally, if possible, researchers should pre-register their study.

## Recommended Gaze Variables to Measure

In most cases, researchers should decide on important AOIs in the referee's visual field and measure dwell times and number of dwells to AOIs. This can be difficult in dynamic situations where these AOIs move and specialized algorithms or software are required. For example, in gymnastics, AOIs can include the tablet or notebook in which scores are recorded, or the gymnast's lower body and upper body. These AOIs move when the gymnast moves and when the head of the judge moves.

In dynamic and fast-moving sports (e.g., association football, ice-hockey), referees should attend to players' movements across large areas, and they must use saccades to shift their gaze between several locations. In such conditions, researchers should also measure (regardless of specific AOIs) the number and duration of fixations and saccades, and saccade amplitudes. As mentioned earlier, the ability to measure saccades accurately depends, among other things, on the sampling rate of the eye tracking system.

Although eye-tracking technology has improved significantly over recent years, obtaining reliable results from wearable eye trackers is often overestimated (Hessels et al., 2020). Researchers should keep this in mind when attempting to record gaze behavior in naturalistic settings, and include the eye-tracking technology they use in their decisions on study objectives and design.

## Can We Generalize Knowledge from One Sport to Another?

We suggest that in a number of sports referees' gaze behavior is probably similar, and hence it is possible that results from a study on one sport can be generalized to another. For example, games like ice hockey and association football require referees to constantly move and position themselves in locations where they can view the action. Gaze behavior in both sports should include fixations on relevant AOIs (where the action takes place, for example).

In contrast, it is unlikely that the gaze behavior of softball umpires will be relevant to the gaze behavior of ice hockey or association football referees. Softball umpires are mostly stationary and are required to see details, such as the ball location as it passes home plate or whether the ball reaches the glove of an infielder before the foot of the base runner touches the base. This requires fixating within a relatively small AOI and is different from the larger visual fields that football or ice hockey referees need to cover. However, gymnastics judges and softball umpires may share some commonalities. Like umpires in softball or baseball, judges in gymnastics look at a small AOI—the athlete itself—and attend to small details (e.g., did the legs stay straight during a jump?).

In summary, in cases where the job description of referees is similar—if, for example, in both sports the referees are mostly stationary (or mobile), and if in both sports the visual field is narrow (or wide)—transfer of knowledge between the sports remains possible.

## Practical Implications—the Missing Link

Due to the small number of studies (*n* = 12), as well as to the diverse findings of these studies (see Table 3), practitioners should adopt a careful approach if they plan to implement gaze-training programs for referees. Indeed, five of the reviewed studies reported differences in both performance and gaze behavior between referees, but in four studies referees of a higher skill outperformed referees of a lower skill without an apparent difference in gaze behavior. We can explain the contradictory findings by (a) the differences in environmental conditions and tasks, (b) the differences in defining “expert” and “novice” referees—an expert referee who makes few decision errors is not necessarily the referee with the most experience, but experience (rather than performance) is often used to differentiate experts from novices, (c) the differences in the way referees interpret the visual information they attend to, and (d) the unknowns regarding the role of team or shared gaze behavior.

Regardless of the underlying reasons, data are insufficient to suggest how gaze behavior assists skilled referees to perform better than their less-skilled counterparts. Additional knowledge should be gained before gaze training can be appropriately and effectively used in programs designated to improve the gaze behavior of referees in sport.

Practitioners who work with referees are advised to take into account evidence-based knowledge when developing training programs aimed at facilitating improved gaze behavior. Presumably, such training programs can benefit the referee in his or her visual attention and decision-making processes that take place in actual competitions/games. However, considering all the findings of our review, we maintain that at this stage of inquiry, evidence-based knowledge on gaze behavior in referees in sport will mainly assist researchers, but not practitioners, in their future work.

## Author Contributions

GZ and RL wrote the first draft. GZ, SZ, and SB conducted the literature search. SZ, SB, and WH edited and contributed to the final version of the manuscript. All authors critically reviewed and contributed to the manuscript.

## Conflict of Interest

The authors declare that the research was conducted in the absence of any commercial or financial relationships that could be construed as a potential conflict of interest.
